# Is there an inhibitory-response-control system in the rat? Evidence from anatomical and pharmacological studies of behavioral inhibition

**DOI:** 10.1016/j.neubiorev.2009.07.003

**Published:** 2010-01

**Authors:** Dawn M. Eagle, Christelle Baunez

**Affiliations:** a Department of Experimental Psychology, University of Cambridge, Downing Site, Cambridge CB2 3EB, UK; b Laboratory of Neurobiology of Cognition, CNRS UMR6155, Aix-Marseille Université, Marseille, France

**Keywords:** Dopamine, Serotonin, Noradrenaline, Atomoxetine, Orbitofrontal, Subthalamic nucleus, Dorsomedial striatum, Nucleus accumbens, SSRT, Premature response, Perseverative response

## Abstract

Many common psychiatric conditions, such as attention deficit/hyperactivity disorder (ADHD), obsessive-compulsive disorder (OCD), Parkinson's disease, addiction and pathological gambling are linked by a failure in the mechanisms that control, or inhibit, inappropriate behavior. Models of rat behavioral inhibition permit us to study in detail the anatomical and pharmacological bases of inhibitory failure, using methods that translate directly with patient assessment in the clinic. This review updates current ideas relating to behavioral inhibition based on two significant lines of evidence from rat studies:

(1) To integrate new findings from the stop-signal task into existing models of behavioral inhibition, in particular relating to ‘impulsive action’ control. The stop-signal task has been used for a number of years to evaluate psychiatric conditions and has recently been translated for use in the rat, bringing a wealth of new information to behavioral inhibition research.

(2) To consider the importance of the subthalamic nucleus (STN) in the neural circuitry of behavioral inhibition. This function of this nucleus is central to a number of ‘disinhibitory’ disorders such as Parkinson's disease and OCD, and their therapies, but its role in behavioral inhibition is still undervalued, and often not considered in preclinical models of behavioral control.

Integration of these findings has pinpointed the orbitofrontal cortex (OF), dorsomedial striatum (DMStr) and STN within a network that normally inhibits many forms of behavior, including both impulsive and compulsive forms. However, there are distinct differences between behavioral subtypes in their neurochemical modulation.

This review brings new light to the classical view of the mechanisms that inhibit behavior, in particular suggesting a far more prominent role for the STN, a structure that is usually omitted from conventional behavioral-inhibition networks. The OF–DMStr–STN circuitry may form the basis of a control network that defines behavioral inhibition and that acts to suppress or countermand many forms of inappropriate or maladaptive behavior.

## 1 Introduction

Without an ability to inhibit behavior or action, it would be impossible to perform even the simplest of everyday tasks. During complex activities such as driving a car, behavior may be stopped, reviewed or changed, perhaps many times within a single minute. Behavior may need to be stopped because it is inappropriate in a particular situation (for example accelerating at traffic lights if the light is red), although the same response might be appropriate elsewhere (when the light turns green), or if there is competition with other possible actions in a set of programmed behaviors (for example accelerating versus braking).

In a broad sense, behavioral inhibition can be viewed as a critical executive-control mechanism that regulates a wide range of cognitive and motor processes with one unified outcome—to prevent the execution of an action. Although this concept of behavioral/motor, inhibition has attracted the interest of psychologists and neuroscientists for many years (reviewed in Aron, 2007), it is only within the last decade that its neural basis has been studied in great detail, and the recent special issue of *Neuroscience and Biobehavioral Reviews* (vol. 33(5), April 2009) on stopping brings this evidence together. This interest has, in part, been directed by clinical studies showing that pathological or maladaptive levels of inhibition failure are common to a number of neuropsychiatric conditions such as attention deficit and hyperactivity disorder (ADHD), Parkinson's disease, schizophrenia, obsessive-compulsive disorder (OCD), chronic substance abuse (e.g., cocaine, amphetamine, methamphetamine), and pathological gambling or shopping (Aron, 2007; Aron and Poldrack, 2005; Bellgrove et al., 2006; Durston et al., 2008; Fillmore and Rush, 2002; Fillmore et al., 2002, 2006; Gauggel et al., 2004; Monterosso et al., 2005; Nigg et al., 2004; Oosterlaan et al., 1998; Penades et al., 2006; Rubia et al., 1998, 2007, 2005; Schachar et al., 2007, 1995; van den Wildenberg et al., 2006). Indeed, there is now a wealth of evidence to suggest that these behavioral impairments can also be useful markers of genetic risk factors for many of the disorders mentioned above (e.g., Aron and Poldrack, 2005; Congdon et al., 2008; Durston et al., 2008, 2006; LeMarquand et al., 1999; Menzies et al., 2007; Nigg et al., 2004). For example, deficient motor inhibition has always been considered as one of the key executive function deficits within an integrative model of the ADHD spectrum (e.g., Castellanos et al., 2006), and test batteries that include measures of inhibition (e.g., stop-signal, go/no-go and delay-discounting tasks) have been used with great success to assess this condition, both in children and adults (Boonstra et al., 2008; Rubia et al., 2007; Sergeant et al., 2003).

Harnishfeger (1995) further defined behavioral inhibition as the control of overt behavior such as motor inhibition, resisting temptation, delay of gratification and impulse control. In the context of neuropsychiatry, these features of behavioral inhibition are most commonly studied in terms of their failure. Suboptimal inhibition is considered to be a critical component of many psychiatric symptoms including impulsivity, compulsivity, perseveration, disinhibition, obsessions, aggression, attention deficits and mania (Aron, 2007). Thus, while there is normally a balance between behavior and its inhibition that allows us to live and function well, the breakdown of behavioral inhibition mechanisms, in conditions such as those listed above, can result in behavior that is maladaptive or inappropriate. Of particular relevance to this review, behavioral inhibition failure may lead to actions that are ‘impulsive’ (rapid or without adequate planning or forethought, carried out without regard to the negative consequences of these actions), or that are ‘compulsive’ (where the repeated performance of a behavior continues despite adverse consequences, often as part of a ritual or addiction). Both of these forms of behavioral inhibition failure have been investigated extensively in rat studies.

In the clinic, complex test batteries have been designed to include a range of measures of behavioral inhibition. These simple tests, such as the go/no-go and stop-signal tasks, also have the advantage of being easily translated between human and rodent studies without the need for significant changes in experimental design. Therefore, these tests provide a strong framework for cross-talk between clinical and preclinical research during investigation of the neural basis and experimental therapeutics of particular disorders.

Often, these tests are used interchangeably and convergently to assess deficient behavioral inhibition in patients. However, within preclinical research, this unitary concept of behavioral inhibition has become outdated (Cardinal et al., 2004; Evenden, 1999; Winstanley et al., 2006), following studies, such as that of Evenden (1999), which examined sub-types of behavioral inhibition in the context of impulsivity in particular. Indeed, impulsivity is often defined primarily as ‘a lack of behavioral inhibition’, including actions that are premature, mistimed, difficult to suppress and control, and also including impulsive choice, where actions are initiated without due deliberation of other possible options or outcomes (Dalley et al., 2007b). Furthermore, Evenden (1999) suggested that these subtypes of impulsivity, or inhibitory deficit, might arise from the dysfunction of different fundamental anatomical and neurochemical mechanisms. For example, different neural substrates may underlie the dichotomy of ‘impulsive action’ (the inability to inhibit a prepotent response) versus ‘impulsive choice’ (the selection of a small, immediate reward in favor of a larger, but delayed, reward) (Cardinal et al., 2004; Winstanley et al., 2006).

In parallel, ‘impulsivity’ has been compared with other forms of behavioral inhibition failure, for example, ‘compulsivity’ (recent examples include: Arzeno Ferrao et al., 2006; Belin et al., 2008; Chamberlain et al., 2006a; Chudasama et al., 2003b; Grant and Potenza, 2006; Potenza, 2007). The clinical relevance of this work is highlighted by addiction research that proposes a transition from impulsive to compulsive behavior during progression from recreactional drug use to addiction (Belin et al., 2009).

Despite numerous lines of evidence to the contrary, many clinical studies still define a unitary concept of behavioral inhibition using a wide range of diagnostic tasks interchangeably in its evaluation. One aim of this review is to highlight the pitfalls of such a unitary approach strategy and the limitations that this approach brings to understanding the neural basis of behavioral inhibition mechanisms and their failure. For example, in many clinical studies, the stop-signal task is considered as an equivalent measure of motor inhibition to the go/no-go task, in particular when assessing impulsive action control. However, the stop-signal task measures the *speed* at which an *already-engaged* response is inhibited, whereas the go/no-go task measures the *ability* to inhibit the *initiation* of a response. Therefore, the go/no-go task may contain response-selection and ‘waiting’ elements that are absent from inhibition in the stop-signal task, and that are subserved by different neural mechanisms. Indeed, recent evidence has highlighted both anatomical and pharmacological differences between these inhibitory processes (Eagle et al., 2008b; Rubia et al., 2001).

In this review we have re-examined aspects of the anatomical and pharmacological basis of behavioral inhibition in the rat in the context of two major new lines of evidence. Firstly, we integrated evidence from the recently developed stop-signal task for rats with existing models of impulsive action (5-choice serial reaction time (5-CSRT) task) and impulsive choice (delay-aversion/delay-discounting task) that have been used extensively in our laboratories (these tasks will be presented in greater detail in the following sections). The stop-signal task is a well-validated model of behavioral inhibition that has been used for many years in clinical studies, of ADHD in particular. As we have said, this task assesses the *speed* of the process of inhibition (stop-signal reaction time, SSRT) of an ongoing action. SSRT is widely accepted within the psychological literature as a unique and indisputable form of motor inhibition (MacLeod et al., 2003) that is increased/impaired in conditions that show symptomatic deficits in behavioral inhibition (Boonstra et al., 2005b; Logan, 1994; Logan and Cowan, 1984; Oosterlaan et al., 1998). For example, recent reviews have indicated that SSRT is a critical and fundamental component of impulsive-action inhibition (Aron, 2007; Dalley et al., 2007b; Eagle et al., 2008b; Pattij and Vanderschuren, 2008). However, there are aspects of its modulation within the brain that seriously bring to question the validity of impulsive action as a single construct.

**Fig. 1:**
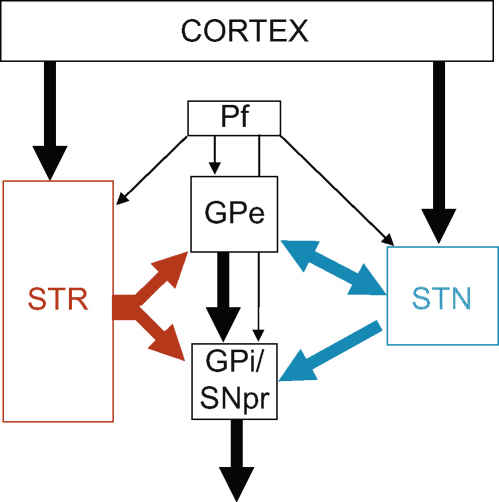
Schematic representation of the model of the basal ganglia proposed by Levy et al. (1997). Str: Striatum; Pf: parafascicular nucleus of the thalamus; GPe: External segment of the Globus Pallidus; GPi: Internal segment of the Globus Pallidus; SNpr: Substantia *nigra pars reticulata*; STN: subthalamic nucleus.

Secondly, we have updated the frontal-basal-ganglia circuitry of behavioral inhibition to include recent evidence for the role of the subthalamic nucleus (STN). The STN is a small cerebral structure that has long been associated with motor processes, since its infarct is known to induce a hyperkinetic-like syndrome, or ‘ballism’ (Whittier, 1947). More recently, the STN has been targeted surgically in the treatment of Parkinsonism (see Benabid, 2003 for review) and this has necessitated a better understanding of its functional involvement in behavioral control. The STN is conventionally thought of as an output structure of the basal ganglia, acting as part of the indirect, potentially inhibitory, cortico-striato-thalamic circuitry (DeLong, 1990). Recently, an updated model of basal ganglia organization highlighted the direct connections between the cortex and the STN (the now so-called ‘hyperdirect pathway’), placing the latter in a position to share, with the striatum, the role of ‘major input structure’ to the basal ganglia from the cortex (Levy et al., 1997) (Fig. 1). This model not only gives more functional importance to the STN, but has also contributed to the STN being seen as a key frontal-cortex target, which therefore should be involved in classical ‘frontal functions’ such as behavioral inhibition. Although a few previous reviews have connected the STN with behavioral inhibition, this structure usually remains missing from, or under-represented within, proposals of inhibitory circuitry (Cardinal, 2006; Dalley et al., 2007b; Robbins, 2002).

Recent anatomical and behavioral studies have proposed that the hyperdirect pathway, between the frontal cortex (possibly the right inferior frontal cortex in humans) and STN, represents a critical route through which information could be processed rapidly. This is supported by evidence that the STN is strongly implicated in the modulation of inhibition on the stop-signal task: a hyperdirect network could maintain the necessary speed of information-processing during this form of inhibition (see Chambers et al., 2009). For example, STN activation correlated with faster stopping abilities (Aron and Poldrack, 2006), and also correlated with activation of the right inferior frontal gyrus (RIFG), a region that has significant associations with stopping (Aron et al., 2003b).

Therefore, from the integration of these new lines of evidence, we propose that regions within the frontal-basal-ganglia network might subserve a general mechanism for behavioral inhibition in the rat. Such a system in the human brain, comprising the RIFG, striatum and STN (or RIFG and STN via a hyperdirect pathway) has received considerable recent interest for its ability to perform rapid inhibition of behavioral responses (for review, see Chambers et al., 2009). The existence of a comparable system in the rat would open up interesting possibilities for translational research into the mechanism of action of behavioral inhibition.

## 2 Translation between clinical and preclinical neuroscience

**Fig. 2:**
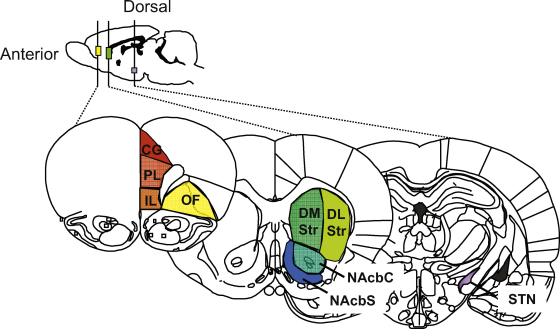
Schematic sections of rat brain showing some of the regions of the cortex and basal ganglia that mediate the control of behavioral inhibition. CG: pre-genual cingulate cortex, PL: prelimbic cortex, IL: infralimbic cortex, OF: orbitofrontal cortex, DMStr: dorsomedial striatum, DLStr: dorsolateral striatum, NAcbC: nucleus accumbens core, NAcbS: nucleus accumbens shell, STN: subthalamic nucleus.

In clinical research, the relative contributions of specific brain regions to behavioral inhibition can be evaluated by observing how behavior is changed in people with brain damage within those regions. In general, patient groups with cortical and basal-ganglia damage show impaired behavioral inhibition compared to healthy control subjects (Aron et al., 2003b; Rieger et al., 2003). For example, Aron et al. (2003b) showed that patients with regional lesions within the RIFG had increased/impaired SSRTs, whereas patients with lesions within adjacent regions, or within the left inferior frontal gyrus had no such impairments. To complement this clinical research, similar behavioral changes can be assessed in rats by directly lesioning or inactivating discrete target regions in the brain. Some of the key regions of the rat brain that are pinpointed in behavioral inhibition research are shown in Fig. 2.

In rat studies, brain inactivation may be permanent (e.g., lesions using glutamatergic excitotoxins such as quinolinic and ibotenic acid), but also may be reversible (e.g., using pharmacological agents to block a particular system [e.g., glutamate antagonists], or to activate an inhibitory system [with GABA agonists such as muscimol]). Other recent studies have used High Frequency Stimulation, which purportedly also blocks activity within a target structure while stimulating the passing fibers (Degos et al., 2005), but has the advantage that its effects can be started and stopped more rapidly. Therefore, in rats, regional inactivation may be targeted precisely to address hypotheses that cannot be investigated with the correlative analysis of human patient studies.

In general, lesions produced in this way are approximately symmetrical in each hemisphere of the rat brain. However, these techniques can be extended beyond the scope of the human lesion studies to investigate connectivity *between* brain structures that are critical to behavioral inhibition. This disconnection can be assessed by lesioning one brain region (e.g., within the cortex) in the left hemisphere and a different region (e.g., within the basal ganglia) in the right hemisphere, and comparing task performance with the effects of bilateral lesions to either brain region alone, or where both structures are lesioned within the same hemisphere. This level of investigation is almost impossible in clinical studies.

There has been much debate about the extent to which such lesion studies in rats can translate to provide useful comparisons with the regional function of the human brain. In summary, direct translation of cortical structural homology between rat and human is controversial (Preuss, 1995), and so it would be unwise to translate any region of the rat prefrontal cortex as a direct structural representation of a region of the human cortex. In particular, the more lateral parts of the human prefrontal cortex are very difficult to compare structurally with rat prefrontal cortex. However, for many years we have used the argument of ‘functional homology’ (see Robbins, 1998) to make tentative comparisons between structures that appear to modulate the same behavioral functions across the species.

In contrast to the cortex, structures within the basal ganglia have largely been conserved in evolutionary terms, making it credible to make direct comparisons between rat and human. For example, the rat dorsomedial striatum is considered to be a functional equivalent of the human caudate nucleus head, with the more lateral part of the rat striatum more closely resembling the human putamen. The more ventral nucleus accumbens in the rat is subdivided into core and shell regions on the basis of different immuno-staining for calcium-binding protein and different connectivity, and the two regions are associated with different functions. Although less documented, these different territories are also present in the human brain (Meredith et al., 1996). The STN in rats and humans is both structurally and functionally comparable (Parent and Hazrati, 1995).

With respect to pharmacological translation, we have found strong similarities between neurochemical manipulations of stop-signal task performance in rats and clinical groups, giving increasing confidence that preclinical studies can contribute to the understanding of clinical pathology. However, such investigations are intrinsically limited by the risk that behavioral changes relating to neurotransmitter/receptor manipulations may be the result of the enhancement/impairment of an inhibitory system, the impairment/enhancement of a behavioral-activation system, or a combination of both. Therefore, it is often difficult, without further study, to determine the precise mechanisms underlying the neurochemical control of behavior.

In this review, we have tried to identify commonalities and differences in the neural circuitry and/or pharmacology underlying different forms of behavior and their inhibition in the rat, in a way that will have direct clinical relevance. To this end, the behavioral tests central to this review have been developed either directly from clinical versions of diagnostic tests, or have contributed to their development. Therefore, there are clear translational implications of the findings of this review for clinical research and the development of novel therapeutic strategies for disorders with prominent inhibitory control deficits. In particular, underlying anatomical or pharmacological differences between subtypes of inhibition may explain why drugs that are effective at treating inhibitory deficits in some people are ineffective in others. This may be relevant to heterogeneous disorders, such as ADHD, that comprise several diagnostic symptoms with inhibitory components, e.g., impulsivity and inattentiveness. A clear challenge for future research is to determine the degree of separation or overlap in the pathology of these disorders, in order to provide the most effective treatment regime in each case.

## 3 Evaluation of behavioral inhibition in the rat

### 3.1 The stop-signal task

The stop-signal task assesses the *speed* of the process of inhibition in an action that has already been initiated. The critical measure on this task, SSRT, is the time taken to stop a response, from the point at which a ‘stop-signal’ is presented, to the point at which inhibition is completed. SSRT is typically of the order of a few hundred milliseconds, but is significantly increased in disorders such as ADHD, which means that these people take longer to stop themselves from completing an action when instructed to do so, and critically, may fail to stop themselves in time before the action has been carried out.

**Fig. 3:**
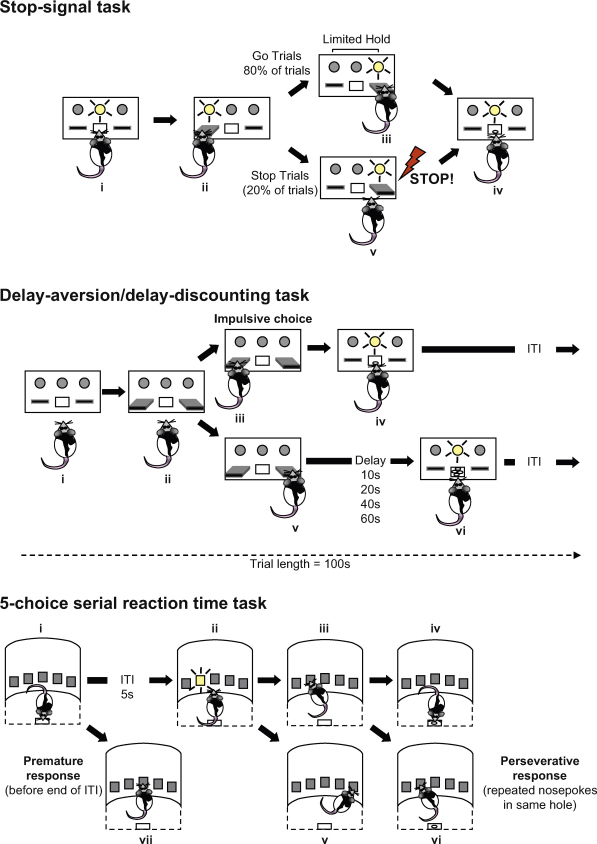
Schematic representation of (a) the stop-signal task, (b) the delay-discounting task and (c) the 5-CSRT task. Each figure shows the functional panels from operant-conditioning chambers (a and b) and the 9-hole, or 5-hole box (c). In the stop-signal task, rats begin each trial with a nose poke in the central food magazine (i). The go trial phase begins with a left lever press (ii) and then the rat must move quickly to press the right lever (iii) to complete the ‘go’ response. A correct trial is rewarded with a food pellet (iv). On 20% of trials (randomly distributed through the session), a stop signal during the go phase signals that the rat must inhibit the right lever press (v) to receive a food pellet. In the delay-aversion/delay-discounting task, trials begin automatically (i) with presentation of both levers (ii). Selection of one lever (e.g., the left lever, iii) gives one food pellet with no delay (iv). Selection of the other lever (e.g., the right lever (v) gives four pellets but after a delay of 0, 10, 20, 40, or 60 s (vi). The rats receive an inter-trial interval (ITI) for the remainder of each 100-s trial to ensure that rats completing no-delay trials do not earn greater numbers of rewards simply by completing greater numbers of trials. In the 5-CSRT task, the rat begins each trial with a nose poke in the food magazine (i), which is located on the opposite wall of the chamber to the response apertures. Following a 5-second ITI a brief (500ms) light appears in one of the apertures (ii) and the rat must make a nose poke response in that hole (iii) to receive a food reward (iv). Responding in a different hole is incorrect (v). Perseverative responding is measured as repeated nose poke responses after the food has been delivered (vi). Impulsive action is measured as premature responses, where a response occurs during the ITI (vii) before the light signal.

All stop-signal tasks are based on the same fundamental principles. A subject performs a rapid reaction time response on a majority of trials, the prepotent ‘go’ response. On a small proportion of trials, a stop signal is presented at a pre-designated point during the course of the response and subjects must attempt to inhibit their response. The closer the subject is to completing the response, the more likely it will fail to inhibit. In our version of the rat stop-signal task, rats are trained to respond rapidly between the left and right levers in an operant-conditioning chamber (Fig. 3). This is the go response. On occasional trials, rats are presented with a brief auditory stop signal (tone) to instruct them to stop this response. The closer the rat is to completing the right lever press, the more likely it is that the rat will fail to stop in time.

SSRT cannot be measured directly because there is no observable endpoint to the response inhibition. Instead, SSRT is estimated using a well-designed, and rigorously tested mathematical model, the race model, developed by Logan and others (Logan, 1994; Logan and Cowan, 1984; also, see Verbruggen and Logan, 2009 for review). If stop-signals are presented close to the endpoint of the response, the stop and go processes race for completion. An earlier stop signal will result in response inhibition, and a later stop signal might allow the go process to complete first, and the response will be executed. An estimate of the endpoint of the stop process (and therefore SSRT) can be integrated from the proportion of successfully inhibited trials and the distribution of go-trial reaction times.

However, in order for the race model to apply, subjects must comply with a set of performance constraints. They must attempt to respond as quickly as possible on go trials (to prevent response slowing in anticipation of a stop signal, which would improve stopping performance), and must always attempt to stop following a stop signal. It is possible, with respect to stopping in particular, that there are occasions where subjects do not detect the stop signal, or where response selection has been made independently of (and probably before) their assessment of trial type (stop or go). For this reason, it is important to assess as many facets of stop-signal task performance as possible before estimating SSRT, as this will determine the suitability of the data for application of the race model. In the rat stop-signal task, every effort is made to check the data for the types of inhibitory deficit that fall outside the assumptions and constraints of the race model, and preclude its application. In clinical studies, these checks are often omitted, so care should be taken when interpreting SSRT-related deficits in these cases.

### 3.2 The 5-choice serial reaction time (5-CSRT) task

The 5-CSRT task was originally developed to assess visuo-spatial sustained and divided attention. The version of this task used for the rat was based on Leonard's test used in human studies (Robbins, 2002). Rats are trained to respond to a brief visual stimulus, presented in one of five apertures, by making a nose-poke in that aperture. However, the rat must first wait, or withhold responding, for a fixed (typically 5 s) or variable inter-trial interval (ITI), while scanning the apertures for the stimulus. Therefore, it is possible for rats to make an ‘impulsive action’ by responding prematurely with a nose-poke before the end of the ITI, i.e., before the stimulus light is illuminated.

Recent interest in impulse-control disorders such as ADHD has increased the number of studies using the 5-CSRT task primarily to measure ‘premature’ and ‘perseverative’ response inhibition deficits. Premature responding is used as an index of the ability to ‘wait’, or withhold a planned, prepotent response. In this respect it is remarkably similar in form to premature responding in tests such as the differential reinforcement of low rates of responding (DRL), in which rats must withhold from responding on a rewarded lever until a fixed time has elapsed (O’Donnell et al., 2005; Pattij et al., 2003). Although the 5-CSRT task can be used to study both rat and mouse behavior, it should be noted that premature responding is far more commonly seen in rat, than in mouse, studies.

In addition, the 5-CSRT task measures perseverative responding, a possible form of compulsive action, when a rat continues to respond by poking its nose into an aperture after the first response (correct or incorrect) has been made, even though these responses are no longer appropriate and have no further positive consequence. Perseverative responding in the 5-CSRT task has similarities with perseverative deficits in other tasks such as the progressive ratio (PR) task, in which rats are required to make a number of lever presses to achieve reward, but where the lever press response may be repeated to excess, even following the completion of the required lever presses and at the expense of reward collection. These perseverative responses may be considered compulsive if compulsion is defined as an inability to inhibit the repetition of prepotent actions, in particular, if the level of perseveration has negative consequences (by reducing the number of achievable rewards in a session, and even more so if perseverative actions are punished by removal of reward delivery on a correct, but actively perseverated response). Thus, the 5-CSRT task provides a practical method of assessing two forms of behavioral inhibition failure that are commonly studied using other tests, and that may also translate to behavioral measures in clinical studies.

### 3.3 The delay-aversion (delay-discounting) task

Delay-discounting tasks are often used as measures of reward evaluation, given the choice between a small immediate reward and a larger, but delayed, reward. In our version of this task, rats are trained to choose between two levers: choosing one lever delivers one food pellet immediately, whereas choosing the other lever delivers four pellets. Over the course of a session, the delay between response and 4-pellet delivery is increased (e.g., 0, 10, 20, 40 and 60 s). Impulsive choice corresponds to a higher likelihood of choosing the smaller reward when the delay for the larger reward increases, even though it is economically advantageous to choose the bigger reward (Cardinal et al., 2001; Evenden, 1999; Evenden and Ryan, 1999). This task is not only interesting from the perspective of assessing impulsive decision making, but it also allows us to address the issue of reward-driven actions because this task introduces competition between impulsivity and motivational processes. For example, an inhibition failure may express itself as an increased choice of one reward over the other, whatever the consequences of that response; as Damasio (1994) called a “myopia for the future” (taken from Winstanley et al., 2006).

## 4 Behavioral inhibition in the stop-signal task

### 4.1 Lesion studies

**Fig. 4:**
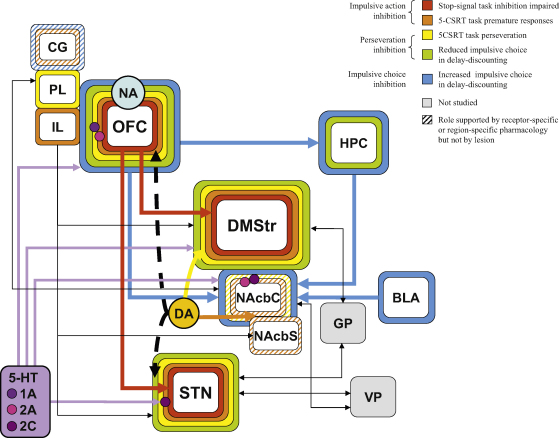
Schematic representation of the major inhibitory processes in the stop-signal, 5-CSRT and delay-discounting tasks from lesion and pharmacological manipulations. Colored bands surrounding each structure highlight the roles of key structures in inhibitory processes. Hatched-shaded bands indicates no effect of excitotoxic lesions but an effect under other circumstances (e.g., dependent on previous behavior, or following pharmacological manipulations). Grey structures indicate that no information is available. Arrows highlight connections between regions that are of interest for response inhibition networks.

Through regional excitotoxic lesion studies of the cortex and basal ganglia, a possible SSRT-mediating neuroanatomical network has emerged that is remarkably consistent across several measures of behavioral control (described in later sections). Lesions within the orbitofrontal cortex (OF), dorsomedial striatum (DMStr) and subthalamic nucleus (STN) disrupt inhibition on the stop-signal task (see Table 1 and Fig. 4). OF lesions and DMStr lesions produced clear effects on SSRT (the speed of the inhibition process). OF lesions impaired/increased SSRT in the absence of any effects on the prepotent go response. DMStr lesions also increased SSRT, but this was accompanied by a slowness to perform the go trials (increased and more variable GoRTs) (Eagle et al., 2008c; Eagle and Robbins, 2003a). Although STN lesions impaired stop-signal task performance, this was expressed as a failure in the *ability* to inhibit on stop-signal trials, more strongly indicative of a generalised attentional or response selection (no-go-like) deficit (Eagle et al., 2008c). More specifically, STN lesions impaired stopping even when the stop signal was presented early in trials (and where subjects should be able to stop with 100% accuracy). Such impairments fall outside the constraints of Logan's race model. However, this behavioral effect was so extreme that it may have masked any effects of STN lesions on the more subtle measure of SSRT and further work is required to extract a clear representation of STN-lesion effects on SSRT in rats.

These results directly translate to human studies in which regions of the cortex, the striatum and the STN are implicated in SSRT modulation (Aron, 2007; Aron et al., 2003b; Aron and Poldrack, 2006; Li et al., 2008a,b; Rieger et al., 2003; Rubia et al., 2001, 2003). In addition, increased SSRT (as well as increased/more-variable GoRT) is seen in ADHD as a result of fronto-striatal dysfunction (Boonstra et al., 2005b; Oosterlaan et al., 1998). As we have discussed, although there is no direct anatomical translation between the OF and specific regions of the human cortex, the OF in rats has such strong functional comparability to the RIFG in relation to stop-signal task performance that this homology is worth further investigation.

The role of the human STN in stop-signal inhibition is particularly intriguing. fMRI studies consistently correlate STN activity with performance on stop-signal trials rather than go trials, giving clear evidence that the STN plays an important role in the *ability* to stop (replicating findings from the rat study) (Aron and Poldrack, 2006; Li et al., 2008b). Furthermore, a hyperdirect pathway between cortex and STN could provide the rapid information-processing necessary for the fast inhibition found in the stop-signal task (Aron and Poldrack, 2006; Chambers et al., 2009). However, there is conflicting evidence with respect to the relationship between STN function and SSRT itself (it should also be noted that these studies did not consider the potential for STN-related changes in performance that fall outside the constraints of the race model). One set of studies correlated high STN activation with short SSRTs (Aron and Poldrack, 2006) and also linked STN activation with that of the RIFG, a region that has strong associations with SSRT control (Aron et al., 2003b). However, another study found opposing results, correlating high STN activation with long SSRTs, instead finding that subjects with short SSRT had higher levels of activation in the caudate (Li et al., 2008b). This finding perhaps fits better with evidence from Parkinson's disease, where the STN is reported to be hyperactive, and where SSRT is increased, suggesting an inhibitory deficit (Gauggel et al., 2004). High frequency stimulation of the STN, but not surrounding structures, in these patients improves SSRT (van den Wildenberg et al., 2006). This also suggests that the particular role of STN in stopping might be influenced by the dopaminergic system integrity. Thus, the STN is undisputedly implicated in inhibitory control in the stop-signal task, but we still have much to learn about its precise function in behavioral control.

Perhaps of greatest significance to our synthesis of an inhibitory-response network in rats is the *lack* of effect of lesions to the prelimbic cortex (PL), infralimbic cortex (IL) and nucleus accumbens core (NAcbC) on SSRT. These regions are anatomically adjacent to the OF and DMStr (Eagle et al., 2008c; Eagle and Robbins, 2003b), and are critical to other forms of executive control (Dalley et al., 2004; Robbins, 1996, 2007). This evidence suggests there is a regionally discrete cortico-basal-ganglia-circuitry specific to SSRT control. However, there is still much to learn about SSRT-mediating circuitry in the rat. For example, the roles of the cingulate cortex (CG) and pallidum remain to be tested in the stop-signal task. The CG in particular may be an interesting target for investigation on the stop-signal task in rats. While perhaps not likely to affect SSRT *per se*, this region may contribute to error detection during task performance, a feature of the anterior cingulate cortex in both ADHD and healthy subjects (Chevrier et al., 2007; Li et al., 2007a,b; Liotti et al., 2005).

### 4.2 Pharmacological studies

#### 4.2.1 DA

The main clinical evidence for pharmacological control of SSRT comes from trials of potential treatments for ADHD, in particular the psychostimulants methylphenidate and d-amphetamine. Both drugs improve/decrease SSRT in most cases, although some reports suggest they fail to affect SSRT in up to 30% of patients (Aron et al., 2003a; Boonstra et al., 2005a; de Wit et al., 2000; Tannock et al., 1989a). Indeed, their action may be baseline dependent, improving SSRT most dramatically in subjects with high (i.e., impaired) baseline SSRTs, but having little or no effect in subjects with low baseline SSRTs (Boonstra et al., 2005a; de Wit et al., 2000; Eagle and Robbins, 2003a; Eagle et al., 2007; Feola et al., 2000). Additionally, these drugs may be non-selective and often decrease the reaction time on go trials (GoRT) (Bedard et al., 2003; Lijffijt et al., 2006; Tannock et al., 1989b), actually potentiating other forms of impulsive action, as well as having other undesirable behavioral side-effects.

Although the psychostimulants are considered to exert their effects primarily through the action of dopamine, there is only limited evidence that SSRT is influenced by dopamine receptor manipulation. There is certainly no general role for dopamine receptors in the modulation of this form of behavioral inhibition, since systemic treatment with the dopamine reuptake inhibitor GBR-12909 had no effect on SSRT (Bari et al., 2009). Additionally, systemic treatment with the mixed D1/D2 receptor antagonist, cis-flupenthixol, failed to affect SSRT, and perhaps more critically, had no effect on the SSRT-decreasing effects of either methylphenidate or modafinil (an atypical stimulant), even at doses that significantly increased GoRT (Eagle et al., 2007). Nevertheless, evidence is starting to emerge that DMStr DA-receptor subtypes modulate SSRT, which reinforces, yet again, the importance of this brain region in inhibitory control. Direct infusion of D1- and D2-receptor antagonists (SCH 23390 and sulpiride) into the DMStr had opposing effects on SSRT, with SSRT decreased following D1R antagonism and increased following D2R antagonism (Eagle et al., 2008a). We propose that there is D2R-mediated inhibition (most probably *en route* through the indirect pathway to the STN), that might oppose D1R-mediated disinhibition. However, it is not clear if the DMStr represents the only critical site within which dopamine mediates inhibition via its action at D2 receptors, and which is balanced by dopamine at D1-receptors within the same structure. There is some evidence that human D2/D3 receptor availability in the ventral striatum might link with SSRT performance (London et al., 2007), but neither D1R nor D2R antagonist infusions directly into the rat NAcbC had any effect on SSRT, or any other behavioral measure on task (Eagle et al., 2008a). Clearly, more evidence is required in this exciting area of study before we can fully assess the role of dopamine in the modulation of SSRT. This line of further study may be particularly relevant to Parkinson's disease (PD), a disease that results from a disruption of dopamine function. PD patients have longer SSRTs (Gauggel et al., 2004), supporting a role for this modulatory system in the inhibitory processes required for SSRT performance, either directly or indirectly.

#### 4.2.2 5-HT

In addition to their DAergic effects, psychostimulants may also act via NA and 5-HT (Arnsten and Dudley, 2005; Gainetdinov et al., 1999; Kuczenski and Segal, 1997). However, there is no evidence to date that 5-HT plays any significant role in SSRT control. This is surprising, given strong evidence linking 5-HT with other forms of inhibition (see following sections), in particular to control inhibition in the go/no-go task and premature responding in the 5-CSRT task (Harrison et al., 1997a, b, 1999). In both human and rat studies, 5-HT depletion (altered tryptophan diet (ATD) and intracerebroventricular (i.c.v.) 5,7-dihydroxytryptamine (5,7-DHT) lesions respectively) did not alter SSRT or any other primary measure on the stop-signal task (Clark et al., 2005; Eagle et al., 2009), and the selective serotonin reuptake inhibitor (SSRI) citalopram, had no effect on SSRT in either species (Bari et al., 2009; Chamberlain et al., 2006b; Eagle et al., 2008b). Furthermore, 5-HT transporter knockout mice were no different from wild-type controls in any baseline stop-signal task measure (Hausknecht et al., 2006). There are no studies of 5-HT receptor subtype manipulations on this task, and this approach might be worthy of study if 5-HT, like DA, modulates SSRT differently via different receptor subtypes.

#### 4.2.3 NA

Interest in the role of NA in behavioral inhibition has arisen from evidence that psychostimulants may influence impulse control disorders via their effects on NA in the prefrontal cortex (e.g., Arnsten and Dudley, 2005). The focus of noradrenergic modulation of SSRT has been two non-stimulant drugs, the selective NA reuptake inhibitor (SNRI) atomoxetine, and the atypical stimulant modafinil. These drugs decrease/improve SSRT in both rat and clinical studies (Chamberlain et al., 2007, 2006b; Chamberlain and Sahakian, 2007; Eagle et al., 2007; Robinson et al., 2008; Turner et al., 2004, 2003). Atomoxetine is of particular interest because, unlike the conventional stimulant treatment drugs for ADHD, it does not change other aspects of task performance. Also, its action is independent of baseline SSRT, and thus may prove a more effective treatment than the stimulant drugs for a larger proportion of the ADHD population.

Although further research is required to define any locus of NA-specific inhibitory control in the rat, the OF could be a potential target for the NA-dependent improvements in SSRT at least. NAergic modulation of OF could be critical to the function of a general behavioral-inhibition mechanism. Certainly, in clinical studies, atomoxetine activates the RIFG, which is a key structure in the control circuitry of SSRT (Chamberlain et al., 2009), and which has functional similarities to the rat OF in terms of its influence over SSRT.

## 5 Comparing stop-signal task inhibition with other models of impulsive action

Now that the neural mechanisms underlying SSRT behavioral inhibition are becoming clear, it is important to see how these mechanisms compare with those involved in the inhibition of other behavioral processes. We predict that the OF–DMStr–STN circuitry could underlie other forms of impulsive-action inhibition as well as SSRT, and that NA, rather than 5-HT might modulate this behavioral control. In fact, the OF–DMStr–STN circuitry is again highlighted, although not exclusively (see Table 1 and Fig. 4). However, the most striking differences between forms of impulsive action lie in their modulation by 5-HT. This evidence is summarized below and brings into question whether it is acceptable to interpret data from different tasks interchangeably as measures of a unitary concept of impulsive-action control.

There are many tests of impulsive action, but the go/no-go task is perhaps most relevant to translational research as it is frequently used to study inhibitory deficits in patients, especially in the field of ADHD research (Rubia et al., 2007; Vaidya et al., 1998). However, within the rat literature, premature response control on the 5-CSRT task has been most widely used as a reliable and reproducible index of impulsive action control and its failure (Dalley et al., 2007b; Pattij and Vanderschuren, 2008; Robbins, 2002). As a result of its extensive use, premature responding on the 5-CSRT task is often taken to represent all impulsive action in rat studies, for example in comparisons with impulsive choice (Dalley et al., 2007b; Pattij and Vanderschuren, 2008; Winstanley et al., 2006). In the following sections we assess if similar mechanisms control SSRT and premature-response forms of impulsive action. We also evaluate SSRT inhibition in relation to go/no-go and other measures of impulsive action control, including simple reaction time (SRT) and DRL tasks.

In go/no-go, a subject must respond on go trials but inhibit that response in no-go trials, so these tasks measure the *ability* to inhibit a prepotent response. The response requirement (go or no-go) is signaled before the subject begins a response, and the subject selects which response to make. In addition to this response-selection process, on no-go trials, the response inhibition must also be maintained to the end of the trial (i.e., the response tendency must be withheld). The behavioral inhibition processes required to withhold responding on no-go trials may be similar to those required to withhold premature responses on the 5-CSRT task, in the sense that an available response must be inhibited for a defined period until either it, or a replacement action is required. Therefore, although the go/no-go task is often used in the clinical setting as an equivalent of the stop-signal task, we predict that go/no-go tasks could have some features in common with response-selection tasks, or those in which waiting is a significant challenge. However, the ‘waiting’ components of no-go and, for example, 5-CSRT tasks have important differences. For no-go, the ‘respond’ command must be inhibited completely, and no-longer needs to be held online, whereas in the 5-CSRT task, the response requirement must be held online for the duration of the waiting period. Therefore, a ‘waiting failure’ on the no-go task is the result of ineffective cancellation of the online representation of response requirement, whereas ‘waiting failure’ in tasks such as the 5-CSRT task is a truer representation of an inability to withhold responding until it is required. This latter group of tasks might require working-memory/timing processing in addition to a capacity to wait.

In SRT tasks, rats are trained to press a lever and sustain this lever press for a variable interval until the onset of a cue-trigger. A premature response corresponds to an early lever release during the variable interval preceding cue onset, which can result from various types of dysfunction such as inability to hold a response (impulsive-like deficit) and perseverating on the pre-potent response (compulsive-like deficit i.e., lever press, lever release, in alternation). In addition, there may be a ‘delay tolerance’ component that is also found in premature responses in the 5-CSRT task, which will be discussed later. In existing studies, task design does not permit these aspects of behavioral inhibition to be dissociated retrospectively. Despite its potential for the study of behavioral inhibition, the evidence from existing SRT tasks is limited because most of the earlier studies did not mention premature responding effects.

### 5.1 Lesion studies of impulsive action

As predicted, lesions within the OF, DMStr and STN impaired inhibition of other forms of impulsive action as well as SSRT. OF lesions significantly increased premature responding on the 5-CSRT task (Chudasama et al., 2003b), although this effect is often misreported. However, in contrast with the stop-signal task, premature responding was also significantly increased by lesions of the IL, and by NMDA receptor blockade directly into the IL (Chudasama et al., 2003b; Murphy et al., 2005). This involvement of the IL in impulsive-action inhibition may be unique to the 5-CSRT task as combined PL/IL lesions did not impair no-go inhibition (Ragozzino et al., 2002; Risterucci et al., 2003). Although combined IL/PL lesions increased premature responding on SRT tasks (Risterucci et al., 2003), further analysis of these premature responses indicated that this deficit may be a failure to time the waiting period correctly, but in addition, the deficit also shared features with a perseverative-like impairment as the duration of lever press during premature trials was so short.

Neither the more dorsal PL nor pre-genual CG played any significant role in impulsive-action inhibition: lesions of pre-genual CG, PL or NMDA receptor blockade directly into the PL had no effect on premature responding in the 5-CSRT task, although more posterior, post-genual CG lesions increased both premature and perseverative responding on this task (Chudasama and Muir, 2001; Chudasama et al., 2003b; Muir et al., 1996; Murphy et al., 2005), and the absence of effect of PL lesions in the combined PL/IL lesion studies is stated above.

The only measure of impulsive action that produced mixed evidence for OF function was the go/no-go task. Although aspirative OF lesions induced behavioral inhibition deficits (Eichenbaum et al., 1983, 1980), other studies failed to find effects of OF lesions on go/no-go tasks. For example, NMDA-induced lesions of the lateral OF did not impair acquisition of an odour-cued go/no-go task, with subjects able to perform no-go inhibitory responses as well as control subjects (Schoenbaum et al., 2002), and there was no impairment following excitotoxic OF lesions in the no-delay condition in the stop-signal task (equivalent to a no-go condition) (Eagle et al., 2008c). Rats with OF lesions were, in fact, better at inhibiting responding in this ‘no-go’ condition, even though they were impaired at stopping when the stop signal was delayed (Eagle et al., 2008c). Schoenbaum and colleagues concluded that OF damage might be more disruptive to no-go performance if rats had pre-learned several series of discrimination tasks, and might be ineffective in disrupting performance if task acquisition took place subsequent to the lesion surgery. Alternatively, the OF may be more critical during the rapid information processing required to stop an ongoing action quickly, and less so for other inhibitory processes that are prominent in the go/no-go task. However, neither of these explanations is particularly well supported at present, and there is clearly a good case for further evaluating the role of the OF in these forms of behavioral inhibition.

Within the striatum, DMStr lesions profoundly increased premature responding on the 5-CSRT task, and the regional specificity of this effect was again confirmed because lesion damage within regions immediately adjacent to the DMStr, in the dorsolateral striatum (DLStr), NAcbC or nucleus accumbens shell (NAcbS) had no effect on premature responding during normal task performance (Christakou et al., 2004; Murphy et al., 2008; Rogers et al., 2001). However, rats with NAcbC lesions tended to increase premature responding following failed trials (Christakou et al., 2004), suggesting the NAcbC might modulate this form of inhibitory response dependent on reward or feedback from previous trial success. DRL tasks have also shown impaired inhibition (i.e., increased premature responding) following NAcbC but not NAcbS damage (Pothuizen et al., 2005). It should also be noted that damage to the DLStr impaired task performance so extensively that it was impossible to evaluate premature response control following these lesions, and following retraining the rats did not exhibit any “impulsive-like behavior” (Rogers et al., 2001). Although the DLStr has clearly defined functions in habit learning, and in the progression from impulsive to compulsive behavior (Balleine et al., 2009; Belin et al., 2009; Everitt et al., 2008) the role of the DLStr, if any, in impulsive action control remains to be fully evaluated, and will be difficult to study because of major confounding effects of other behavioral impairments that follow manipulations within this region. There are studies of effects of DLStr versus DMStr excitotoxic lesions on SRT performance, but premature responding was not reported (Brown and Robbins, 1989; Hauber and Schmidt, 1994).

Excitotoxic lesions of the STN markedly increased most measures of impulsive action, including premature responding in the 5-CSRT task (Baunez and Robbins, 1997) and also significantly impaired no-go-like inhibition in the stop-signal task (under conditions where there was no delay between go and stop signals) (Eagle et al., 2008c). Both unilateral and bilateral lesions of the STN increased premature responding in various forms of reaction time tasks (Baunez et al., 2001, 1995b; Phillips and Brown, 2000), as did microinfusions of either muscimol or NMDA receptor antagonist AP5 into the major output target of the STN, the substantia nigra pars reticulata (Amalric and Koob, 1989; Baunez and Amalric, 1996). STN lesions also increased impulsive action in a DRL procedure (Uslaner and Robinson, 2006). This evidence strongly supports a role for the STN in a general circuit that inhibits impulsive action under normal circumstances. However, in contrast, pharmacological reversible inactivation of the STN with muscimol or STN high frequency stimulation did not increase premature responding, but increased the number of perseverative responses (Baunez et al., 2007; Baunez and Robbins, 1999b). Therefore, it is possible that a more discrete inactivation of the STN itself may have less of an effect on premature responding, although still having a substantial effect on perseverative responding, and this remains open to study.

The hypothesis that impulsive-action control is maintained through cortico-striatal and cortico-STN circuitry is further supported by disconnection-lesion studies, primarily from the 5-CSRT task. In a series of studies, a large prefrontal cortex lesion (with damage within the OF, IL, PL and CG) was made in one hemisphere and a lesion of the DMStr, NAcbC or STN was made in the other hemisphere. These disconnected lesions were compared with unilateral lesions where both structures were lesioned in the same hemisphere. While the unilateral lesions had little effect in any study, the PFC-DMStr and PFC-STN disconnected rats exhibited more premature (and also perseverative) responses (Christakou et al., 2001; Chudasama et al., 2003a), while mPFC-NAcbC disconnected rats only increased premature and perseverative responses after failed trials, similar to the effects of NAcbC lesions alone (Christakou et al., 2004). This again confirms that both the PFC-DMStr and PFC-STN pathways are critical to premature response control. However, the extent of the PFC lesions in these studies was large, and we can only speculate that it was the OF (rather than IL)–DMStr–STN circuitry that was specifically implicated because the OF connects much more strongly with the DMStr than does the IL (Reep et al., 2003; Schilman et al., 2008; Voorn et al., 2004). The IL instead connects strongly with the NAcbS (Laubach and Woodward, 1995), and although lesions within the NAcbS do not increase premature responding *per se*, there is evidence from pharmacological studies (see below) that this structure may act as the output of impulsive action control from the IL. It may be reasonable to predict that both IL-NAcbS and OF-DMStr disconnection lesions would produce comparable levels of impulsive-action disinhibition in terms of premature responding on the 5-CSRT task. There is also recent evidence of direct connectivity between the OF and STN (Maurice et al., 1998). A challenging prospect for further study is whether the PFC-STN disconnection effects above represent a disruption of the indirect pathway between OF and STN, or are representative of a hyperdirect pathway between these two structures.

### 5.2 Pharmacological modulation of impulsive action

#### 5.2.1 5-HT

Pharmacologically, the greatest difference between SSRT and premature responding as measures of impulsive action lies in their modulation by 5-HT. Although there is little evidence that 5-HT influences SSRT, premature response control on the 5-CSRT task is closely linked with 5-HT function. For example, premature responding correlates with a high level of 5-HT turnover (Puumala and Sirvio, 1998) and transgenic rats lacking the serotonin transporter show fewer premature responses (Homberg et al., 2007). This suggests that 5-HT may act as a brake in the control of impulsive responses, perhaps enhancing the ability to wait. In contrast, 5-HT depletion, obtained by intraventricular 5,7-DHT infusion, increased premature responses (Carli and Samanin, 2000; Harrison et al., 1997a; Winstanley et al., 2004b). This central/global effect is probably mediated at the level of the dorsal striatum, based on the following evidence. 5-HT-depleting lesions of the dorsal raphé nucleus (that reduced 5-HT predominantly in the dorsal and ventral striatum and cortex) significantly increased premature responding on the 5-CSRT task, whereas lesions of the median raphé nucleus (that reduced 5-HT predominantly in the dorsal and ventral hippocampus and cortex) had no effect (Harrison et al., 1997b). Neither mPFC nor NAcb 5-HT depletion reproduced a premature response increase (Fletcher et al., 2009), which implicates, by subtraction, the dorsal striatum (and we predict the DMStr) in the 5-HT-mediated control of this form of impulsive behavior. 5-HT also modulates impulsive action deficits in other tasks, for example, premature responding in DRL and go/no-go tasks (Dekeyne et al., 2002; Eagle et al., 2008b, 2009; Evenden, 1998; Fletcher et al., 2009; Harrison et al., 1999; Higgins et al., 2003; Liao and Chang, 2001; Marek et al., 2005; Masaki et al., 2006; O’Donnell et al., 2005; Pattij et al., 2003). Global 5-HT depletion following i.c.v. infusions of 5,7-DHT profoundly disrupted the acquisition of no-go inhibition, and also impaired the ability of previously-trained rats to subsequently inhibit correctly to a no-go signal (Harrison et al., 1999), with no change in other task measures. Similarly, rats administered with parachloroamphetamine, to induce 5-HT depletion in the brain, were slower to acquire no-go inhibition (Masaki et al., 2006). In the stop-signal task, although SSRT was not affected by 5-HT depletion, rats were less able to withhold responding on stop-signal trials if they had to withhold for an extended period (similar to the increased ITI test in the 5-CSRT task) (Eagle et al., 2009). This further dissociates ‘waiting’ and SSRT, suggesting that 5-HT is far more important in controlling the former.

This influence of 5-HT over premature response control appears to be receptor-subtype-dependent: the action of 5-HT at 5-HT2C receptors opposes, and at 5-HT2A receptors increases, premature responding. For example, systemic treatment with the 5-HT2A receptor antagonist M100907 decreased premature responses in control (Fletcher et al., 2007), but not in 5-HT-depleted, rats (Winstanley et al., 2004b) and also decreased premature responding on DRL (Higgins et al., 2003). In contrast, the 5-HT2C receptor antagonist SB 242084 increased premature responding in all rats, both in 5-CSRT and DRL tasks (Higgins et al., 2003; Winstanley et al., 2004b; Fletcher et al., 2007), and the 5-HT2C receptor agonist WAY-163909 decreased premature responding in normal rats (Navarra et al., 2008a).

This receptor-subtype specificity may be peculiar to premature response control, since neither 5-HT2A nor 5-HT2C drugs affected perseverative responding in normal and 5-HT-lesioned rats on this task (Robinson et al., 2008). It may also be predominantly basal-ganglia-mediated because when these 5-HT-receptor-subtype-specific drugs are infused directly into regions of the basal ganglia, they produce remarkably similar effects to the systemic effects. For example, infusions of the 5HT2A receptor antagonist M100907 into the NAcb decreased premature responding (Carli et al., 2006) but the 5HT2C receptor antagonist SB242084 increased premature responding (Robinson et al., 2007). This is particularly intriguing given the lack of effect of 5-HT depletion in the NAcb on premature response control, and implies that, within the NAcb, 5-HT2A and -2C receptor subtypes oppose one another to modulate the action of 5-HT in impulse control.

Furthermore, 5-HT2C receptors appear to have the same modulatory function at the level of the STN because the 5-HT2C receptor agonist Ro 60-0175, either systemic or infused directly into the STN, abolished the premature responding induced by muscimol infusion into the STN (Baviera and Carli, 2007). This suggests that throughout the basal ganglia, 5-HT2C receptor activity could play an important role to modulate 5-HT2A-receptor-mediated premature responding. It is not currently known if 5-HT receptors have similar roles within the DMStr.

A slightly different balance of 5-HT-receptor-subtype control is seen within the PFC. 5-HT2A receptors are again implicated in premature response modulation. However, perhaps surprisingly, there is little evidence that 5-HT acting at 5-HT2C receptors is able to oppose/suppress premature responding, and this is an important area for further study. For example, the 5-HT2A/C receptor antagonist ketanserin reduced premature responding when infused into the mPFC (Passetti et al., 2003), and the 5HT2A receptor antagonist, M100907, reduced CPP-induced premature responses (Carli et al., 2006). In addition, the 5-HT1A receptor may not be critical to premature response control because the 5-HT1A receptor agonist, 8-OHDPAT, had no effect on this measure in the 5-CSRT task (Carli et al., 2006). Since a recent study has shown that 5-HT2A receptor antagonists had no significant effect on premature responding when infused into the IL or PL (Robinson et al., 2007), it is possible that the region in which 5-HT2A receptors play their critical role to modulate premature-response impulsivity is the OF (Fig. 4).

#### 5.2.2 DA

DA is considered to be an important modulator of impulsive action because psychostimulants generally improve SSRT. However, psychostimulants have the opposite effect on impulsive action in the 5-CSRT task, and generally increase/worsen impulsivity (increase premature responding). The DA reuptake inhibitor GBR 12909 also has this effect, suggesting that inhibitory control failure in the 5-CSRT task is the result of increased DAergic transmission (van Gaalen et al., 2006). In particular, the NAcb has been highlighted as a key structure here, despite the relative lack-of-effect of lesions within this structure on premature responding. For example, impulsive rats selected on the basis of individual performance (i.e., exhibiting a higher level of premature responding in the 5-CSRT task) have reduced availability of D2/D3 receptors in the NAcb (Dalley et al., 2007a). In addition, there is direct evidence for psychostimulant action in the NAcb: intra-accumbens amphetamine dose-dependently increased premature responses (Cole and Robbins, 1987) to the same extent as systemic injections of amphetamine (Cole and Robbins, 1989). Furthermore, there is a dissociation of function within the NAcb itself. Although lesions of the NAcbC and NAcbS do not affect premature responding *per se*, d-amphetamine-induced premature responding was increased following NAcbC lesions, but decreased following NAcbS lesions, which suggests “functionally opposed or co-modulatory roles” for these subregions in premature response control (Murphy et al., 2008).

The D1 receptor is a possible target for this psychostimulant action, since the intra-NAcb-amphetamine impulsivity could be blocked by systemic D1/D2 mixed receptor antagonism (cis-flupenthixol) and the D1 receptor antagonist SCH23390 also reduced premature responses when administered systemically (Harrison et al., 1997a; van Gaalen et al., 2006). In addition, the D1 receptor agonist SKF38393 increased premature responding when administered directly into the NAcb (Pezze et al., 2007). Although these drugs were infused in the ventral portion of the NAcbC, the authors acknowledge that they cannot discount the possibility that they may have also acted within the NAcbS, so it is not possible to attribute these effects, definitively, to DA in the NAcbC alone. Conversely, neither D2-receptor antagonist sulpiride nor eticlopride affected premature responding, via systemic or intra-accumbens routes (Pezze et al., 2007; van Gaalen et al., 2006). Nevertheless, although D2-receptor antagonism had no effect on premature responding in isolation, it reduced stimulant-induced premature responding, indicating “competitive antagonism of the effects of drugs of abuse on premature responding through blockade of DA D2 receptors” (van Gaalen et al., 2006).

The action of DA at specific receptor subtypes in the dorsal striatum is unclear. It is surprising, given the marked effects of DMStr lesions on premature responding, that few pharmacological manipulations have been made within this structure. Therefore, any conclusions about the role of striatal DA in impulsive-action control can only be based on DA depletion results. We predict that there would be a more prominent role for DA (and possibly D2-receptor-mediated) manipulations in the DMStr that link to behavioral inhibition rather than activation.

Neither DA depletion of the dorsal nor the ventral striatum had any effect on premature responding in the 5-CSRT task (Baunez and Robbins, 1999a; Cole and Robbins, 1989). Nevertheless, there is evidence for opposing roles of DStr and NAcb in premature response control. DA depletion of the ventral striatum *decreased* the number of premature responses induced by systemic amphetamine and DA depletion of the dorsal striatum had the opposite effect and *potentiated* d-amphetamine-induced premature responding (Baunez and Robbins, 1999a). This evidence again reinforces the hypothesis that inhibitory control failure on this task may result from increased DAergic transmission, but that DA transmission in the ventral striatum might activate responding and DA transmission in the DMStr might reduce or oppose responding on this task. From the available evidence it is not possible to determine if DA receptors in the dorsal striatum promote behavioral inhibition, reduce behavioral activation, or both, but there are certainly opposing roles for the dorsal and ventral striatum in the overall control of behavioral output.

During SRT task performance, psychostimulants such as d-amphetamine also increased premature responses (Baunez et al., 1995a), and the duration of the lever presses indicated that amphetamine did not induce a perseverative-like behavior, unlike those observed after mPFC lesions (Risterucci et al., 2003). This suggests that amphetamine-induced premature responses could result from a problem in estimation of time intervals, and this effect of amphetamine might also influence the ability to ‘wait’ in the 5-CSRT task. Further research is required to determine if DA-mediated premature responding is simply a timing-control deficit. Although there is no evidence for the role of the NAcb in this respect, lesions of the DA terminals within the dorsal striatum increased premature responding depending on the extent of the lesion in the SRT task (Amalric et al., 1995; Turle-Lorenzo et al., 2006). However, given that the d-amphetamine-induced effects on this task may have had more of a perseverative than a premature response basis (Baunez et al., 1995a), the effects of dorsal striatal DA manipulations on the SRT task may be more closely comparable with the 5-CSRT perseverative responding deficit observed after DA lesions in the dorsal striatum than with a true premature responding (Baunez and Robbins, 1999a).

#### 5.2.3 NA

The importance of cortical NA function in behavioral inhibition in the 5-CSRT task has recently been confirmed. Although NA depletion had no direct effect on impulsive action in the 5-CSRT task (Carli et al., 1983; Cole and Robbins, 1992; Milstein et al., 2007), low doses of the noradrenaline reuptake inhibitors desipramine or atomoxetine reduced premature responding (the opposite effect of dopamine reuptake inhibitors), in both normal rats (Navarra et al., 2008b; Robinson et al., 2008; van Gaalen et al., 2006) and rats selected as ‘impulsive’ on the basis of their individual performance in the 5-CSRT task (Blondeau and Dellu-Hagedorn, 2006). These studies are of interest for further research as atomoxetine also improved SSRT in rats (and both atomoxetine and desipramine improve SSRT in people), suggesting that NAergic drugs might be more predictable to control impulsive actions than DAergic drugs.

Research into the importance of NA-receptor subtypes to behavioral inhibition has been limited, although all of these studies support a role for NA to modulate inhibition. For example, the alpha-2a receptor agonist guanfacine decreased premature responding, both in normal and NA-depleted rats (Milstein et al., 2007). More interestingly, with respect to behavioral inhibition and ADHD, the beta-adrenoceptor antagonist, propranolol antagonised a methylphenidate-induced increase in premature responding on the 5-CSRT task (an effect that was confirmed as brain-mediated rather than peripherally-mediated because the peripheral beta-adrenoceptor antagonist, nadolol failed to have such an effect) (Milstein et al., 2008). This suggests that methylphenidate is exerting its effects on behavioral inhibition at least partly through NA receptors, most probably in the cortex, as well as DA receptors in the striatum. Although there is no direct evidence to place these NA-receptor effects within any distinct region of the cortex, we predict that the OF and IL would be good targets, based on their roles in impulsive action control that is revealed by lesion studies, and from clinical studies of NAergic drugs such as atomoxetine, that act within specific regions of the cortex during inhibitory control (Chamberlain et al., 2009).

#### 5.2.4 Acetylcholine

Lesions of the cholinergic system performed with either excitotoxins or selective 192-IgG Saporin failed to affect either premature or perseverative responses (see review by Robbins, 2002). However, under certain circumstances (increased stimulus duration or increased inter-trial interval, repeated administration), nicotine can increase premature responses (Blondel et al., 1999; Mirza and Stolerman, 1998; van Gaalen et al., 2006). The role of acetylcholine in impulsive action control does not seem to be major.

## 6 Comparison of ‘perseverative’ and ‘impulsive’ behavioral inhibition

Perseverative/compulsive responding is considered to represent a very different form of behavioral inhibition deficit from impulsive action. However, perseverative response control has many underlying similarities with impulsive-action control, as supported by evidence from the 5-CSRT, switching/reversal and progressive ratio (PR) tasks, that have apparently perseverative performance errors (i.e., a change in response that requires termination of one action and adoption of a new, correct action, but where choice of the ‘old’ action persists). This evidence again supports a role for the OF–DMStr–STN circuitry in a general behavioral countermanding system (Fig. 4).

Lesions within OF, DMStr and STN all significantly increased perseverative responding in the 5-CSRT task, whereas lesions within IL, CG and DLStr failed to have an effect (Baunez and Robbins, 1997; Chudasama and Muir, 2001; Chudasama et al., 2003b; Rogers et al., 2001). Of the cortical regions investigated in 5-CSRT task studies, only PL lesions produced significant levels of perseverative responding outside of this OF–DMStr–STN network. Both OF and PL lesions markedly increased perseverative responding on the 5-CSRT task (Chudasama et al., 2001, 2003b) and resulted in the appearance of perseveration deficits in other tasks. For example, OF lesions severely impaired reversal learning (Boulougouris et al., 2007; McAlonan and Brown, 2003, although not attentional set-shifting (McAlonan and Brown, 2003). Similar findings were reviewed by Ragozzino (2007), who summarised the work from a number of studies of prefrontal and medial striatal contributions to reversal and switching behavior. Most prominent was the action of the OF during reversal, where OF inactivation specifically produced perseveration of a previously correct response in a two-choice test, in the absence of any sampling of a now-correct but previously incorrect option. The PL, on the other hand, had a prominent role in switching tasks, signifying its importance in abstract rule application. Again these deficits were perseverative. However, PL inactivation did not induce any deficits in reversal learning, unlike OF inactivation. Therefore, although both OF and PL are critical to circuitry that modulates perseveration, each contributes to the inhibition of subtly different forms of this impairment. Critically, OF- and PL-lesion-related perseverations appear to result from a failure to sample alternative strategies or options. In particular, the OF may support processes that enable the ability to shift away from a previously relevant choice, given changes in outcome. However, a recent study (Tait and Brown, 2007) showed that OF-lesioned rats were more likely to follow the reversal of the rule if the newly rewarded stimulus was novel (with the previously rewarded stimulus remaining the same), but less likely to reverse if the newly rewarded stimulus was the same and the other (newly unrewarded) stimulus was novel. They suggest that pure perseveration cannot account for all of the OF deficits in reversal, and postulate that learned non-reward is also an important function of the OF.

Perhaps surprisingly, the IL, which is critical to premature response control, appears to play no role in the control of perseverative responding, for example, on the 5-CSRT task (Chudasama et al., 2003a, b). Similarly, Ragozzino (2007) summarised that IL-related performance error in reversal/switching was the result of failure to adopt new strategies rather than an inability to inhibit a previously correct strategy.

Again disconnection lesions between the cortex and basal ganglia supported a regional specificity and discrete connectivity underlying perseverative response control that was essentially the same as the circuitry defining premature-response inhibition (although again, the PFC lesions produced in these studies cannot separate roles for PL, IL, OF and CG without further study). Rats with PFC-DMStr disconnection made more perseverative responses in the 5-CSRT task (Christakou et al., 2001; Chudasama et al., 2003a), while the PFC-NAcbC disconnected rats showed more perseverative responding only after failed trials (Christakou et al., 2004). Disconnection between PFC and STN profoundly increased perseverative responses (Chudasama et al., 2003a), which may support the existence of a functional hyperdirect pathway connecting the cortex and STN, although further studies would need to confirm this. These results strongly suggest that similar cortical-basal-ganglia projections could inhibit both inappropriate impulsive and perseverative responses, more prominently via the PFC-DMStr than the PFC-NAcbC route.

We therefore predict that the DMStr and STN should have the same role as the OF and PL in controlling perseverative responding. Indeed, inactivation of DMStr impaired perseverative response control in the 5-CSRT task (Rogers et al., 2001), while lesions of the DLStr and NAcbS had no effect and NAcbC lesions increased perseverative responses only after failed trials (Christakou et al., 2004; Murphy et al., 2008). DMStr lesions also impaired reversal and switching (Ragozzino, 2007). However, in the case of reversal/switching, this deficit was not purely perseverative, since the striatally impaired rats were able to shift responses away from the previously correct response to the newly correct response, but still made significantly more choices of the previously correct response. DMStr lesions also increased perseverative lever pressing in a progressive ratio (PR) task, such that the rats continued to respond on the lever for a significant number of lever presses after the reinforcer had been delivered (Eagle et al., 1999). A similar effect may have been seen following neonatal lesions of mPFC because although these lesions impaired PR breakpoint, they also increased the overall number of lever presses made (Schneider and Koch, 2005). Unfortunately, the role of the OF on PR performance is unclear, although we would predict comparable OF and DMStr function during perseverative response control on this task.

DMStr-related perseveration can occur even when the rats have sampled all of the available response options (Tait and Brown, 2007). This suggests that the DMStr may play a much broader role in behavioral flexibility than either the OF or PL alone, perhaps permitting the complete inhibition of one or more options from a set of possible responses in the light of information about the other available strategies or actions. This is not inconsistent with other views about the role of the striatum in behavioral control.

As predicted, STN disruption profoundly impaired the control of perseverative responding in the majority of studies. Only one study to date has found evidence that may contradict our predictions, showing that STN-lesioned rats exhibited a better behavioral flexibility than sham control rats in reversal learning, although no actual measure of perseverative behavior was reported (El Massioui et al., 2007). However, in the 5-CSRT task, bilateral lesions of the STN markedly increased perseverative responses, both in the apertures and in the magazine where the reward was collected (Baunez and Robbins, 1997). Pharmacological reversible inactivation of the STN with muscimol or STN high frequency stimulation also increased perseverative responding (Baunez et al., 2007; Baunez and Robbins, 1999b). In the PR task, bilateral lesions of the STN also increased perseverative lever presses, accompanied by increased PR breakpoint for food (Baunez et al., 2002). Interestingly, these perseverative lever presses were diminished, as well as the PR breakpoint, when the reward was cocaine after STN lesions (Baunez et al., 2005), which shows again the importance of reward value to modulate perseverative-like behavior.

### 6.1 Pharmacology of perseverative response control

#### 6.1.1 5-HT

Normal 5-HT function is implicated in the suppression of perseverative responses (as well as suppression of premature responding) on the 5-CSRT task, and thus, 5-HT depletion resulted in increased perseverative responding on this task (Harrison et al., 1997a,b; Winstanley et al., 2004b). 5-HT-receptor-subtype contributions to the modulation of perseverative responding are complex and possibly region-specific. However, unlike premature response control, the 5-HT1A receptor is strongly implicated in the inhibition of perseverative responding because the 5-HT1A agonist, 8-OHDPAT, reduced CPP-induced perseverative responding when infused directly into the mPFC (Carli et al., 2006). There is also evidence from a reversal task for 5-HT2A and -2C receptor involvement at a cortical level, since perseverative errors decreased following infusions of the 5-HT2C-receptor antagonist SB 242084 into the OF, and instead 5-HT2A antagonism (with M100907) increased perseverative responding (Boulougouris et al., 2007).

However, in the NAcb, neither 5-HT2A nor -2C antagonism affected perseverative responding (Robinson et al., 2007). It is not known if these regional differences in 5-HT2A- and -2C-receptor effects on perseverative responding represent fundamental behavioral differences between tasks, or truly represent regional differences in receptor subtype function. Systemic SB 242084 failed to affect perseverative responding, either in 5-HT-depleted or sham-operated rats (Winstanley et al., 2004b), suggesting perhaps that regional 5-HT2C-receptor opposition of function might be a possibility, but that the locus of these effects is unclear and should be examined further. Additionally, it would be interesting to examine the role of 5-HT1A receptor agonists at the level of the striatum to determine if the 5-HT1A receptor is critical to perseverative response suppression throughout the cortico-striatal circuitry. Such region-specific opposition of effects appears across different neurotransmitter systems and is a fascinating prospect for further research, especially considering the possible relevance for progressive degenerative disorders that may cause disruption of function in some regions of the brain before others.

#### 6.1.2 DA

In the 5-CSRT task, dorsal striatal DA depletion significantly increased perseverative responses under standard test conditions (Baunez and Robbins, 1999a), showing that DA is critical for perseverative-response inhibition. By perseverating in responding, rats fail to switch to the next step of the session, and this might relate to the shifting problems described in parkinsonian patients. This effect might involve specifically the D2 receptor. The only evidence for DA-receptor specific modulation of perseveration, on the tasks we have considered in this review, comes from direct infusions of DAergic drugs into the NAcb, which showed that the D2 agonist quinpirole significantly increased perseverative responding (Pezze et al., 2007), whereas neither a D2-receptor agonist nor any D1-receptor manipulations in this region had an effect. We speculate that this D2-receptor-mediated perseveration could be suppressed by an opposing effect of DA at D2 receptors in the DMStr.

#### 6.1.3 NA

Very few studies have considered the role of NA in perseverative responding, although there is some promising evidence that NAergic drugs may improve perseverative impairments in reversal-learning tasks. Although lesions of the dorsal noradrenergic bundle had little effect on reversal learning (Tait et al., 2007) noradrenaline reuptake inhibitors, such as atomoxetine and desipramine improved reversal performance in rats, apparently as a result of decreased perseverative errors (Seu et al., 2008).

## 7 Neuroanatomy and pharmacology of impulsive choice on the delay-discounting task

### 7.1 Decreased choice of large reinforce—impulsive choice?

In the delay-discounting task, ‘impulsive choice’ is defined as the less-profitable choice of a small, immediate reward over a larger reward that is delayed. Lesion studies suggest that the neural mechanism that suppresses inappropriate levels of impulsive choice may be different from that which suppresses impulsive and compulsive action. Lesions of the NAcbC increased choice for a small immediate reward on the delay-discounting task (Cardinal et al., 2001). This was considered as an impulsive choice related to delay aversion rather than a decrease in reward magnitude perception, since NAcbC-lesioned rats preferred the larger reward to the smaller when both were presented with no delay to reinforcer delivery. Basolateral amygdala (BLA) lesions (Winstanley et al., 2004a) and extensive lesions of the hippocampus (HPC) (Cheung and Cardinal, 2005) produced comparable effects to those of NAcbC lesions, also resulting in impulsive choice. This evidence suggests a BLA–NAcbC–HPC network that provides control over impulsive choice and sensitivity to delay.

Within the prefrontal cortex, PL/IL or anterior CG appeared not to contribute to the control of impulsive choice (Cardinal, 2006). Within the basal ganglia, NAcbS lesions also had no effect on this form of inhibition (Pothuizen et al., 2005). Once again, this reinforces the anatomical specificity of circuitry controlling behavior, this time for impulsive choice, albeit through a different network to other forms of impulse control discussed so far.

### 7.2 Increased choice of large reinforcer–perseverative choice or delay tolerance?

In contrast to the network mentioned above, Winstanley et al. (2004a) found that OF lesions induce increased choice for the large-but-delayed reward when compared to the performance of control animals, implying that the OF might promote impulsive choice, and perhaps opposing a hypothesis that the OF forms part of an inhibitory network. However, other studies, such as Mobini et al. (2002), found that lesions of the OF induced the opposite effect of apparent impulsive choice in a similar task. Once again the role of the OF in inhibitory control may be complex. If the delay discounting task has a critical goal-directed component, this between-study discrepancy may result from a difference in the size of the larger reward (1 versus 4 and 1 versus 2 respectively), and therefore the degree of contrast between the small and large reward. The role of the OF in reward-related behavior is well-known and can account for the effects described by Winstanley et al. (2004a). Indeed, it would favour the hypothesis that impulsive choice is under the control of the outcome in OF-lesioned animals. At a neuronal level, Roesch et al. (2007) found that although neuronal firing in the rat OF was strongest in response to immediate rewards, some neurons displayed sustained firing in anticipation of delayed reward. Therefore, it is possible that different processes within the OF govern its role in the selection of impulsive choice or delay tolerance. For this reason, differences between tasks, or regional differences in lesion damage within the OF may bias towards very different behavioral profiles in delay-discounting tasks.

We can also hypothesise that the differences in apparent delay tolerance are the result of perseverative choice towards the larger reinforcer, even when this reinforcer is delayed. This would be specifically pronounced for within-session increases in delay to the larger reward, such that early session choice of the large reward persisted as delay grew larger. This hypothesis, therefore, predicts that lesions to structures that are critical to perseverative response control on other tasks might also induce apparent delay tolerance in a delayed-reinforcement task. Indeed, although lesions of both the STN and DMStr increased choice for the large-but-delayed reward in this task (Winstanley et al., 2005, Eagle et al., unpublished data) a recent intertemporal choice study has indicated little role for the STN in delay discounting (Bezzina et al., 2009). Each of these structures, as well as the OF, is also strongly involved in the assessment of reward value (for review on the OF see Everitt et al., 2007; for STN and reward value see Baunez et al., 2005; also electrophysiological studies have revealed a specific encoding of reward value by STN neurons, Lardeux et al., unpublished data). This OF–DMStr–STN circuitry may therefore modulate a number of behaviors through its control of perseverative responding that have previously been associated with other subcategories of inhibitory control. However, despite this perseverative component, there is evidence that DMStr lesions do indeed result in some degree of delay tolerance (Eagle et al., unpublished data), and further investigation of the roles of the OF and STN in this respect would be interesting.

### 7.3 Pharmacology of impulsive choice

#### 7.3.1 DA

Psychostimulants often have opposing effects on impulsive choice, either increasing choice for the delayed reinforcers (Richards et al., 1999; Sagvolden et al., 1992; Wade et al., 2000), or increasing impulsive choice (Charrier and Thiebot, 1996; Evenden and Ryan, 1996). These differences were discussed by Cardinal et al. (2004). Amphetamine increased choice of the small, immediate reward if the large-but-delayed reward was not signalled, but increased the choice for the larger reward if it was signalled (Cardinal et al., 2000). This was hypothesized to result from the well-known effect of amphetamine to enhance conditioned reinforcers, since a signal during a delay to reinforcement would generally increase the rate of responding during the delay, and thus promote choice of the delayed reward (Cardinal et al., 2004). Systemic amphetamine further increased the choice for large-but-delayed reward in STN-lesioned rats (Uslaner and Robinson, 2006), supporting this hypothesis, since STN lesions can also increase reactivity to conditioned reinforcers (Baunez et al., 2002).

In another line of evidence, rats exhibiting impulsive choice had reduced DA release in the NAcbC, NAcbS and mPFC (Diergaarde et al., 2008). These results highlight a role for the DA inputs to those structures that control impulsive choice. However, the precise regions involved in this DAergic action within the PFC should be further specified since lesion studies so far have not suggested any important role for medial PFC regions in this behavior. Again the OF might be predicted to be the most likely prefrontal cortical candidate to modulate this form of impulse control.

#### 7.3.2 5-HT

5-HT depletion leads to impulsive choice in a variety of paradigms, suggesting that normal 5-HT function is critical for appropriate inhibition of impulsive choice (Bizot et al., 1999; Mobini et al., 2000a; Mobini et al., 2000b; Wogar et al., 1993). There are, however, different or opposite effects described in the literature. Stimulating the 5-HT system with the agonist fenfluramine decreased impulsive choice (Poulos et al., 1996). However, Dalley et al. (2002) showed that disinhibition could be associated with elevated 5-HT levels in the mPFC, suggesting that at least at the level of the mPFC, a depletion in 5-HT might not necessarily induce impulsive choice. Furthermore, Winstanley et al. (2003) found no effect after 5-HT depletion in the delay-discounting task. In his review on impulsivity, Evenden (1999) suggests that the influence of 5-HT may depend on the receptors involved and further research could clarify this issue.

#### 7.3.3 NA

To date, only one study has investigated the role of the noradrenaline reuptake inhibitor, atomoxetine, in the delay-discounting task, showing atomoxetine to significantly increase choice for the larger-but-delayed reward (Robinson et al., 2008). This is unlikely to have been a perseveration-related artifact because atomoxetine decreases, rather than increases, perseverative errors on other tasks (Seu et al., 2008). This is the first evidence that NA can modulate inhibitory response control on a number of levels, and indeed this drug is the only one that has been shown to improve inhibition on the stop-signal task, 5-CSRT task and delay-discounting/delay-aversion tasks together, making it a good candidate for a general ‘inhibition-improving’ treatment.

## 8 Is there an inhibitory control system in the rat?—Concluding remarks

In recent years, our understanding of the neural basis of inhibitory control has made significant progress, both in terms of neuroanatomy and neuropharmacology. These studies have reinforced many aspects of the non-unitary nature of behaviour and its inhibition, in particular being defined by differences in neurochemical mechanisms of control. Nevertheless, this review has highlighted that neural circuitry comprising OF, DMStr and STN may be central to the inhibition of many aspects of behavior, in particular acting to suppress, or countermand, inappropriate/maladaptive levels of behavior with ‘impulsive-action’ and ‘perseverative-compulsive’ components. This circuitry may therefore form the basis of a general behavioral inhibition network in the rat brain (Fig. 4).

Such a ‘countermanding’ network, including RIFG, STN and striatum, has already been proposed for the human brain. The existence of such a control network that translates across species presents exciting possibilities for research into therapies that target behavioural inhibition and its dysfunction. Perhaps one aim for the future is to determine if different components of behavioural inhibition failure result from decreased function within this countermanding network, or as a result of over-activation of the processes that initiate or activate action, or a combination of both.

In particular, this review highlights the close association between OF and STN function. This leads the way for studies that can elucidate the precise nature of an OF-STN relationship, whether by the conventional indirect route through the basal ganglia, or utilising the hyperdirect OF-STN circuitry. It is difficult to speculate on future outcomes of research into the function of the STN in this respect, as its relationship with the OF and DMStr is clearly complex. Perhaps the role of the STN defined in this review brings to question how inactivation of the STN could be beneficial for the treatment of conditions related to maladaptive impulsive/compulsive control, for example, OCD (Mallet et al., 2008). Nevertheless, the STN is apparently as fundamental to many aspects of behavioral inhibition as the more traditionally recognised fronto-striatal circuitry.

In clear contrast, impulsive choice may be controlled by different brain regions including the OF again, but also NAcbC, BLA and HPC. In impulsive choice tests, the OF is again linked to DMStr and STN because lesions within these regions result in an apparent decrease in impulsive choice, or improved tolerance of delay. Whether or not this is a true tolerance of delay or an effect relating to perseverative-like deficits remains to be evaluated. It is also difficult to exclude a reward-related effect in the systematic choice for the larger reward, since both OF and STN are involved in motivational processes and STN lesions, for example, increase motivation for food (Baunez et al., 2002). Again, this is an area of research that merits further consideration. Furthermore, the involvement of the same brain regions in reward processes suggests that perseverative-like behavior, guided by the outcome, may also result in a lack of apparent disinhibition in other measures of behavioral control.

Pharmacologically, modulation of behavioral inhibition is more complex. Despite close anatomical similarities between many forms of behavioral inhibition, subtle differences in neurotransmitter function in different regions are critical to the inhibition/release of different behavioral subtypes. Thus, although ‘impulsive action’ and ‘perseverative-compulsive’ behaviors appear to be inhibited through common neural circuitry, the neurochemistry confirms that, when these behaviors occur, they are distinctly different processes, for example, utilizing different sub-populations of 5-HT or DA receptor. There are also some clear region-specific neuropharmacological differences worth noting. In particular the influence of DA via the NAcb predominantly affects ‘premature-like’ behavior, while its influence at the level of the dorsal striatum (in particular the DMStr) may predominantly affect ‘perseverative-like’ behavior.

Furthermore, the integrity of impulsive action as a single concept is brought into question. Firstly, psychomotor stimulants have opposite effects on different forms of impulsive action, increasing 5-CSRT-task impulsivity but decreasing stop-signal-task impulsivity. Secondly, there are clear differences in the role of 5-HT to control different sub-types of impulsive action that have previously been considered as equivalent during clinical assessment. Although 5-HT has been traditionally considered a critical component of impulsive action control, there is a clear lack of effect of 5-HT in the modulation of SSRT compared with other forms of impulsive action. This is theoretically important as it opposes the long-standing status of 5-HT as a ‘universal modulator of inhibition’.

Instead, noradrenaline emerges as a potentially important modulator of behavioral inhibition. Clearly, there is still too little evidence to assess fully the role of NA in the inhibitory response control network, but important recent findings show that the selective noradrenaline reuptake inhibitor atomoxetine is, so far, unique in its ability to reduce inhibitory deficits in all three of the focal tasks described here (reducing SSRT, premature responding and impulsive choice). Indeed, for premature-response control in tasks such as the 5-CSRT task, a range of NAergic drugs improved impulse control whereas the psychostimulants invariably made impulse control worse. Therefore NAergic drugs may prove more suitable candidates for the treatment of a wide range of impulse-control disorders in patients than the currently favoured psychostimulants, which prove effective in some respects but worsen symptoms in others. In rats, the OF seems a likely prefrontal cortical target for the NAergic action of drugs such as atomoxetine, because of its importance as a modulator of inhibitory output. The RIFG might be the equivalent target in human studies as the efficacy of atomoxetine within this brain region is already known. It would be reasonable to hypothesise that drugs targeted at the noradrenaline transmitter system might be a promising line of treatment of inhibitory disorders.

This summary of behavioral inhibition processes in the rat has significant implications in the clinical setting. In a recent review, Pattij and Vanderschuren (2008) suggested that “when considering alleviating impulsivity to treat a psychiatric disorder, one should be aware of which type of impulsive behavior is manifest in that disorder”. Our interpretation reinforces the importance of considering fundamental processes before categorising groups of tasks together. Most notably, we suggest that the stop-signal and go/no-go tasks must not be used interchangeably, or as directly-comparable measures of impulsive action control, as the pharmacology underpinning behaviour in each task is different. Also, behavioral manifestations such as impulsive choice and delay tolerance, which appear to be opposite ends of the same behavioral spectrum, do not necessarily result from opposite changes within a particular brain region or network, and may represent completely different behavioral processes. Conversely, the inhibition of apparently very different forms of inhibitory deficit, such as impulsive action and perseveration, may be far more similar than previously predicted. We conclude that diagnostic tests in the clinical setting should be considered with greater awareness of the exact processes to which their behavioral outcomes relate, and we recommend the preclinical literature as an invaluable source of information in this respect. This may enable more effective and individually-tailored targeting of drug therapies, in particular within spectrum disorders such as ADHD.

## Figures and Tables

**Table 1:** A summary of the effects of lesion and pharmacological manipulations on inhibitory processes on the stop-signal, go/no-go, 5-CSRT and delay-discounting tasks. Additional information is provided for tasks that may be linked to ‘perseverative’ and ‘premature’ response processing. All processes are discussed in terms of increasing or decreasing inhibition, for consistency. Thus, for example, increased premature responding is presented as decrease in inhibition of premature responding. Empty cells indicate that no information is available.

Manipulation	SSRT	Go/no-go	Inhibition of premature responses, e.g., 5-CSRTT, SRT, DRL	Inhibition of perseverative responses, e.g., 5-CSRTT, PR, reversal/switching (R/S)	Delay discounting
**Lesions**					
CG lesion			5-CSRTT	5-CSRTT	No effect (Cardinal, 2006)
			No effect pre-genual (Chudasama et al., 2003b)	No effect (Chudasama et al., 2003a, b)	
			Decrease post-genual (Muir et al., 1996)	Decrease post-genual (Muir et al., 1996)	
			SRT		
			No effect (Risterucci et al., 2003)		

PL lesion	No effect (Eagle and Robbins, 2003b)	No effect (prepotent) (Eagle and Robbins, 2003a,b)	5-CSRTT	5-CSRTT	No effect
		No effect (equipotent) (Ragozzino et al., 2002)	No effect (trend to decrease) (Chudasama and Muir, 2001)	Decrease (Chudasama and Muir, 2001)	
			SRT	R/S	
			Decrease (IL-PL) (Risterucci et al., 2003)	Decrease (Ragozzino, 2007)	

IL lesion	No effect (Eagle et al., 2008c)	No effect (prepotent) (Eagle et al., 2008c	5-CSRTT	5-CSRTT	No effect
		No effect (equipotent) (Ragozzino et al., 2002)	Decrease (Chudasama et al., 2003b)	No effect (Chudasama et al., 2003b;	
			SRT	R/S	
			Decrease (IL-PL) (Risterucci et al., 2003)	Ragozzino, 2007)	

OF lesion	Decrease (Eagle et al., 2008c)	Increase (prepotent) (Eagle et al., 2008c)	5-CSRTT	5-CSRTT	Increase (Winstanley et al., 2004a)
		No effect (Schoenbaum et al., 2002)	Decrease (but no effect following ITI changes) (Chudasama et al., 2003b)	Decrease (Chudasama et al., 2003b)	Decrease (Mobini et al., 2002)
				R/S	
				Decrease	
				(Ragozzino, 2007; Boulougouris et al., 2007)	
				Increase (Tait and Brown, 2007)	

DMStr lesion	Decrease (Eagle and Robbins, 2003a)	Unclear	5-CSRTT	5-CSRTT	Increase (Eagle et al., Unpub.)
			Decrease (Rogers et al., 2001)	Decrease; (Rogers et al., 2001)	
				PR	
				Decrease –Stable with ratio	
				(Eagle et al., 1999)	
				R/S	
				Decrease (Ragozzino, 2007)	

DLStr lesion			5-CSRTT	PR	
			No effect (but rats were unable to perform task) (Rogers et al., 2001)	Decrease –increased perseveration with ratio (Eagle et al., 1999)	

NAcbC lesion	No effect (Eagle and Robbins, 2003b)	No effect (prepotent) (Eagle and Robbins, 2003a, b)	5-CSRTT	5-CSRTT	Decrease (Cardinal et al., 2001; Pothuizen et al., 2005)
			No effect (trend to decrease) (Christakou et al., 2004)	Decrease after failed trials (Christakou et al., 2004)	
			Decrease (/sham) after failed trials (Christakou et al., 2004)		
			DRL		
			decrease especially for long delays (Pothuizen et al., 2005)		

NAcbS lesions			5-CSRTT	5-CSRTT	No effect (Pothuizen et al., 2005)
			No effect (Murphy et al., 2008)	No effect (Murphy et al., 2008)	
			DRL		
			no effect (Pothuizen et al., 2005)		

STN lesion	No effect (Eagle et al., 2008c)	Decrease (prepotent) (Eagle et al., 2008c)	5-CSRTT	5-CSRTT	Increase (Winstanley et al., 2005; Uslaner and Robinson, 2006)
			Decrease (Baunez and Robbins, 1997)	Decrease (nosepokes)	
			SRT	Decrease (panel push)	
			Decrease (Baunez et al., 1995b; Baunez et al., 2001; Phillips and Brown, 2000)	(Baunez and Robbins, 1997)	
				R/S	
				Improved reversal but no effect on perseveration	
				(El Massioui et al., 2007)	
				PR	
				Decrease (Baunez et al., 2002)	

BLA lesion				R/S	Decrease (Winstanley et al., 2004a)
				No effect (Eichenbaum et al., 1986)	

**Disconnection**					
MPFC (PL + IL)—STN			5-CSRTT	5-CSRTT	
			Decrease (transient) (Chudasama et al., 2003a)	Decrease (Chudasama et al., 2003a)	

mPFC—dorsal striatum			5-CSRTT	5-CSRTT	
			Decrease (Christakou et al., 2001)	Decrease (Christakou et al., 2001)	
			No effect following ITI manipulations (Christakou et al., 2001)		

mPFC—ventral striatum (NAcbC)			5-CSRTT	5-CSRTT	
			Decrease (only after failed trials) (Christakou et al., 2004). Decrease (/sham) after failed trials following ITI manipulations (Christakou et al., 2004)	Decrease (only after failed trials)	
				No effect when variable ITI used (Christakou et al., 2004)	

**Pharmacological treatments**					
Serotonin depletion	No effect (Eagle et al., 2009)	Decrease (equipotent)	5-CSRTT		No effect (Winstanley et al., 2003)
		(Harrison et al., 1999; Masaki et al., 2006)	Decrease		Decrease (Mobini et al., 2000a,b: Wogar et al., 1993; Bizot et al., 1999)
		Decrease following extended LH test in stop-signal task (prepotent) (Eagle et al., 2009)	Global or dorsal raphe depletion		
			(Harrison et al., 1997a,b; Carli and Samanin, 2000; Winstanley et al., 2004b)		
			No effect		
			mPFC or NAcb depletion; median raphe depletion		
			(Fletcher et al., 2009; Harrison et al., 1997b)		

KO serotonin transporter	No effect (Hausknecht et al., 2006)		5-CSRTT		
			Increase (Homberg et al., 2007)		

Citalopram (SSRI)	No effect (Bari et al., 2009; Eagle et al., 2008b)		DRL		
			Decrease		
			(Dekeyne et al., 2002)		

Serotonin receptor manipulations			5-CSRTT	5-CSRTT	Increase
			Increase	Increase	fenfluramine (Poulos et al., 1996)
			(5-HT2A) receptor antagonist M100907 into NAcb/mPFC	5-HT1A agonist 8-OHDPAT into mPFC (Carli et al., 2006)	
			(Carli et al., 2006) or systemic (Winstanley et al., 2004b); 5HT2A/C receptor antagonist ketanserin into mPFC (Passetti et al., 2003; Fletcher et al., 2007); 5HT2C receptor agonist WAY-163909; (Navarra et al., 2008a); 5-HT2C receptor agonist Ro 60-0175 into STN (Baviera and Carli, 2007)	Decrease	
			Decrease	5-HT2C antagonist in NAcb (Robinson et al., 2007)	
			5-HT2C receptor antagonist SB242084 systemic or into NAcb ((DRL)Higgins et al., 2003; Winstanley et al., 2004b; Fletcher et al., 2007; Robinson et al., 2007)	No effect	
			No effect in PL or IL (Robinson et al., 2007)	5-HT2C antagonist systemic (Winstanley et al., 2004b)	
			No effect		
			5-HT2A antagonist into mPFC (Carli et al., 2006)		
			No effect 5-HT1A agonist 8-OHDPAT into mPFC (Carli et al., 2006)		

Dopamine depletion (dorsal: striatum)			5-CSRTT	5-CSRTT	
			No effect (Baunez and Robbins, 1999a)+ STN lesion	Decrease (nosepokes)	
			No effect (significantly less than STN lesion alone (Baunez and Robbins, 1999a)	No effect (panel push) (Baunez and Robbins, 1999a)	
			SRT		
			Depends on the extent: either No effect or Decrease (Amalric et al., 1995; Turle-Lorenzo et al., 2006)		

Dopamine depletion (ventral striatum)			5-CSRTT	5-CSRTT	
			No effect (Cole and Robbins, 1989)	No effect (Cole and Robbins, 1989)	

Dopamine receptor manipulations—D1 and D2	No effect of D1/D2 antagonist (cis-flupenthixol) (Eagle et al., 2007)		5-CSRTT	5-CSRTT	
	Increase		Increase	Decrease	
	D1 antagonist SCH 23390 in DMStr (Eagle et al., 2008a)		SCH23390 (D1 antagonist) in both shell and core of NAcb or systemic (Pattij et al., 2007; van Gaalen et al., 2006)	D2 agonist quinpirole (Pezze et al., 2007)	
	Decrease		Decrease		
	D2 antagonist sulpiride in DMStr (Eagle et al., 2008a)		D1 agonist SKF38398 (Harrison et al., 1997a; Pezze et al., 2007)		
			No effect		
			D2 antagonist in either shell or core of NAcb (Pezze et al., 2007; van Gaalen et al., 2006)		

DA reuptake inhibitor	No effect		5-CSRTT		
	GBR 12909 (Bari et al., 2009)		Decrease		
			GBR 12909 (van Gaalen et al., 2006)		

Dorsal noradrenergic bundle lesion			5-CSRTT	R/S	
			No effect (Cole and Robbins, 1992; Carli et al., 1983)	No Effect (Tait et al., 2007)	

NA receptors	No effect/decrease		5-CSRTT		
	Guanfacine (Bari et al., 2009)		Increase		
			Alpha-2a receptor agonist guanfacine		
			Beta-receptor antagonist propanolol (Milstein et al., 2007, 2008)		

Nbm-Ach saporin lesions			5-CSRTT		
			No effect (low dose); Decrease (high dose) (McGaughy et al., 2002)		

Nicotine (ACh receptor pharmacology)			Decrease		
			When increased Stimulus duration, or ITI in 5-CSRTT, or repeated administration (Blondel et al., 1999; Mirza and Stolerman, 1998)		

d-Amphetamine/methylphenidate/ modafinil	Increase		5-CSRTT		Mixed increase or decrease see (Cardinal et al., 2004) for review
	d-Amphetamine (baseline dependent) (Eagle and Robbins, 2003a; Feola et al., 2000)		Decrease		Increase
	Methylphenidate (baseline dependent) (Eagle et al., 2007)		NAcb, NAcbC (Cole and Robbins, 1987; Murphy et al., 2008)		+STN lesion (Uslaner and Robinson, 2006)
	modafinil (Eagle et al., 2007)		6-OHDA lesions (Cole and Robbins, 1989)		
			(D2 antagonist eticlopride in NAcbC blocks and in NAcbS attenuates this. D1 antagonist SCH23390 has no effect in either shell or core) (Pattij et al., 2007)		
			No effect		
			STN lesions (Baunez and Robbins, 1999a)		
			Increase		
			NAcbS(Murphy et al., 2008)		
			SRT		
			Decrease (Baunez et al., 1995a)		

Atomoxetine (SNRI)	Increase (Robinson et al., 2008)		5-CSRTT	R/S	Increase (Robinson et al., 2008)
			Increase (Robinson et al., 2008; Navarra et al., 2008b; Blondeau and Dellu-Hagedorn, 2006; van Gaalen et al., 2006)	Increase (Seu et al., 2008)	
